# A novel approach to welfare interventions in problem multi-cat households

**DOI:** 10.1186/s12917-019-2183-3

**Published:** 2019-12-03

**Authors:** Kayleigh Hill, David Yates, Rachel Dean, Jenny Stavisky

**Affiliations:** 1 Greater Manchester Animal Hospital, 411 Eccles New Road, Salford, M5 5NN UK; 2 Greater Manchester Animal Hospital, 411 Eccles New Road, Salford, M5 5NN UK; 3 Centre for Evidence-based Veterinary Medicine, School of Veterinary Medicine and Science, University of Nottingham, Sutton Bonington Campus, College Road, Loughborough, LE12 5RD UK; 4 Centre for Evidence-based Veterinary Medicine, School of Veterinary Medicine and Science, University of Nottingham, Sutton Bonington Campus, College Road, Loughborough, LE12 5RD UK

**Keywords:** Animal hoarding, Cats, Veterinary hoarding interventions, Neutering, Feline welfare, Preventative, Multicat, Overpopulation

## Abstract

**Background:**

Thousands of injured, stray and relinquished cats are received at the RSPCA Greater Manchester Animal Hospital each year. A significant and challenging proportion of these cats are confiscated from multicat households by RSPCA Inspectors, due to the owners’ inability to care for them. These households share many characteristics of animal hoarding, including poor owner compliance with suggested welfare improvements and recidivism. The relatively poor adoption potential of animals from such households are a perennial problem for the charity.

The aim of this study was to determine if offering female cat neutering assistance to multi-cat owners significantly improved colony welfare.

**Results:**

Ten multicat households with a history of public complaint to the RSPCA were recruited. An RSPCA veterinary surgeon (VS) initially assessed the overall welfare of each household’s cat population, individual cat welfare and the living environment. All entire female cats aged over 8 weeks were neutered and basic animal care education provided. Follow up visits were completed two and 12 months later to reassess welfare parameters and population numbers.

The total number of cats was 176 across ten households (range 7–33, median 16). All owners consented to having all entire female cats spayed. At the first visit, mean individual cat welfare scores ranged from 5.4–8.7/ 16 across the 10 households, where 16 represented best possible welfare.

Overall household mean welfare scores were significantly improved at both the 2 month and 12 month revisits (*p* = 0.011 and p = 0.01 respectively) when compared to the initial visits. By the end of the study period, three out of the ten households had voluntarily relinquished all of their cats, and overall there was a 40% reduction in the number of cats.

**Conclusions:**

Animal hoarding has previously been an intractable welfare concern with little evidence informing intervention techniques. These results show that positive veterinary engagement on site, focused on preventative care and population control, can yield significant improvement in welfare scoring systems in relatively short timescales. Promptly collecting and neutering all female cats at a site, combined with advice and support, show promise in improving welfare.

## Background

Cat ownership is a common phenomenon, with an estimated 23% (UK and Australia) – 30% (USA) of households owning at least one cat [1–3]. Owning more than one cat is relatively common. Of UK cat-owning households, 39.2% had more than one cat, and there were an average of 2.1 cats per household reported in the USA [1, 2]. However, more than two cats per household is less common; in one study only 5.5% of cat owning households had more than 2 cats [1]. Whilst it is possible for households containing larger numbers of cats which are very successfully managed, this is not universally the case. Additionally, although the exact relationship between owning large numbers of cats and hoarding is unclear, evidence suggests a close link [4].

Hoarding is defined as the pathological accumulation of inanimate objects, typically items of low value or rubbish. It is thought to be associated with mental illness, and attempts to characterise its underlying causes are ongoing [5–7]. Animal hoarding is currently indexed in the Diagnostic and Statistical Manual of Mental Disorders (DSM-V) as a subset of object hoarding, itself currently classified as a type of obsessive-compulsive disorder [8]. However, speculations as to the underlying mechanism and classification of hoarding behaviour are ongoing, and include parallels with addictions, possible roots in trauma or underlying organic illness such as dementia [7, 9]. The exact relationship between object and animal hoarding is unclear; and Animal hoarding has elsewhere been described as “a special variant of hoarding that carries unusual community health risks.” [10]. This definition is currently the subject of debate, as animal and object hoarding appear to have separate risk factors [11–13], although many animal hoarders may additionally hoard objects [12, 13].

Animal hoarding is characterised by the pathological accumulation of animals, often but not exclusively pet species [14]. Hoarded animals are typically found in conditions of squalor, malnutrition and neglect, with evidence of inbreeding, poor socialisation and high rates of infectious disease [15]. Animals are often so physically and psychologically compromised that upon confiscation most require humane euthanasia [15, 16]. One case series described the presence of dead cats in 31.6% of hoarding households, and dead dogs in 47.4% [14]. Pet species such as dogs and cats are most commonly reported, but other species, including farm animals, wildlife and waterfowl, have been hoarded [11, 14, 17].

There are several different definitions of animal hoarding, but the key components are generally agreed to include:

- Accumulation of a large number of animals
- Failure to provide minimum standards of nutrition, sanitation and veterinary care
- Failure to respond to the deteriorating condition of the animals or environment, or to the negative effect on themselves [the owners] and those around them [6]

A fourth point, compulsive acquisition and/ or difficulty in giving up any animals, is sometimes but not consistently included as a signifier [6]. Additional signifiers, such as significant impairment to the owner’s social and executive functions and routine activities of daily life, have also been suggested to be potential signifiers [11].

Aspects of this definition, for example, a “large” number of animals, gives scope for interpretation and varies between and within countries. In the US state of Hawaii the threshold for hoarding is a minimum of fifteen animals, whereas in Michigan it is ten [18]. Other legal bodies purposely do not stipulate a figure, suggesting that hoarding is defined by the number of animals exceeding the owner’s capacity for care. However, in a Brazilian study, owners of twenty or more cats showed psychological parallels to hoarders when compared to owners of one or two cats [4]. This suggests that, whilst it is possible to hoard a relatively small number of animals, people who choose to own unusually large numbers of animals may be at increased risk of becoming hoarders. In addition to the number of animals, hoarders have been characterised by the approach of the owner. There are three possible categories described: overwhelmed caregivers, who often become unable to care for their pets due to a change in circumstance; rescue hoarders, who actively acquire animals and may feel only they can save the animals in their care; and exploiter hoarders, described as having sociopathic tendencies and a need for control [19].

Whilst many hoarding interventions have aimed at addressing the symptoms of the “larger maladaptive situation”, unless the underlying condition is treated, recurrence is almost inevitable [9]. In attempts to address this, some prosecutions in animal hoarding cases have included court-mandated mental health treatment. However, this has proved problematic, as compliance is low, exacerbated by many hoarders’ lack of insight into the impact of their behaviours [20]. Additionally in countries such as America, the hoarder might be required to self-fund such treatment [18, 21].

Much of the animal hoarding work has been carried out in the USA. However, emerging work elsewhere in the world suggests that the phenomenon is widespread [4, 5, 7, 22–24]. A recent systematic review of animal hoarders described this population as “neglected and poorly studied”, particularly in regard to potential interventions [13].

Having a specific caseworker who can develop a relationship of trust with the hoarder has been identified as an important technique to provide continuity and follow-up [25], and avoid an adversarial relationship [9]. It has been suggested by those working tackling such cases that an interdisciplinary approach, where the owner’s needs are also supported may be the key to success [20, 21, 26–28]. In support of this, in a recent study where ongoing counselling and animal care advice were provided alongside ongoing support with animal care advice showed promising results in six cases [26].

Complaints about people keeping large numbers of pets, particularly cats, are commonly made to the RSPCA, the UK body which enforces animal welfare legislation. The RSPCA National Call Centre receives around 1000 calls per year regarding multi-animal (> 10 animals) households which have traditionally been investigated by RSPCA inspectors, who typically will make attempts to help an overwhelmed caregiver, or, where necessary, prosecute. It is unclear to what extent these multi-cat households fulfil the characteristics of hoarding, but they do seem to share characteristics, including the fact that so far traditional strategies have not proved successful. This failure to overall significantly improve animal welfare in the long term appears to be due to high rates of recidivism, and therefore an alternative strategy may be of benefit. In August 2012, the RSPCA Greater Manchester Animal Hospital (GMAH) was designated as the preferred location for the triage of vulnerable animals from four neighbouring RSPCA Inspectorate groups in the North West of England. Since then, more than 18,891 stray and relinquished cats have been triaged and processed at the hospital.

The aim of this study was to trial an alternative form of intervention in cases of multi-cat ownership which had been identified as problematic or potential hoarding households to GMAH, to determine if offering female cat neutering assistance to multi-cat owners significantly improved colony welfare.

## Results

Of the ten owners identified, all agreed to take part in the study and consented to having all of their entire female cats spayed. All of the 2 month revisits were on time (+/− 1 week) except for House 5 which was delayed to an interval of 4 months as it was not possible to contact the owner. The second revisits all took place at 12 months (+/− 2 weeks). The basic descriptors of each household and its cats can be seen in Table 1.

**Table 1:** Overall cat numbers and mean Household Welfare Scores at first visit and revisits (2 and 12 months later).

Multi-cat house number	1	2	3	4	5	6	7	8	9	10
Number of cats Visit 1 (following inspectorate referral)	11	17	15	7	26	10	33	15	21	21
Number of cats Visit 2 (~ 2 months)	0	16	13	7	3^a^	5	32	8	18	17
Number of cats Visit 3 (~ 12 months)	0	18	13	0	0	3	32	7	15	17
Number euthanased after discussion with owner over study period	0	0	0	0	0	0	1	3	2	2
Number died over study period	0	0	0	0	0	1	1	0	1	0
Number of new cats over study period	0	2	0	0	0	0	3	1	0	0
Number of cats already neutered at initial visit	0	15	3	2	2	3	3	1	1	5
Number of females spayed during study	3	2	6	5	8	1	16	6	7	9
Number of pregnant spays	1	0	0	0	2	0	7	3	0	2
Total number of litters in household over period of cat ownership	11	3	3	0	11	2	13	4	5	2
Potential for inbreeding? Yes/No (Y/N)	Y	N	Y	N	Y	N	Y	Y	Y	Y
Number of cats signed over to RSPCA	11	0	0	7	18	6	2	6	3	2
Mean Welfare score Visit 1	6.4	5.4	8.7	5.4	4.3	6.9	5.4	6.0	7.8	9.2
Mean Welfare score Visit 2 (2 months)	NA	4.6	4.9	2.0	4.2^a^	6.8	5.7	2.8	5.9	6.5
Mean Welfare score Visit 3 (12 months)	NA	4.3	4.5	NA	NA	5.5	3.3	3.7	4.8	5.7

*NA* not applicable as voluntarily relinquished all cats

^a^ Revisit was done at 4 months instead of 2 months due to inability to make owner contact

### Cat population data

On the initial visits, there were a total of 176 cats across the ten households, with a median of 16 cats per household (iInterquartile Range (IQR) 12–21). At the 2 month revisit, House 1 opted to have all cats signed over to the RSPCA. The nine remaining households had a total of 119 cats (median = 13, IQR 5.5–16.5). No owners had acquired any new cats by the time of the 2 month revisit. At the 12 month revisit, a further two households had relinquished all of their cats, and the remaining seven households owned a total of 105 cats (median = 15, IQR 0.75–16.5). The median number of cats across all 10 households was 16 at visit 1, 10.5 at visit 2 and 10 at visit 3.

Over the 12 month study period the following fluctuations in population occurred -.

- Eight owners felt that they were not coping with the current number of cats and reduced numbers by signing a total of 55 cats over to the RSPCA. This ranged from 2 to 18 cats, with a median of 6 cats signed over per household. Of these cats, 26/55 (47%) were later euthanased for welfare reasons.
- Household 5 sold eight cats.
- A total of three cats died (over two households).
- One cat present at the first visit in House 2 was a neighbour’s cat, which had returned to the neighbour at subsequent visits
- Two cats went missing from House 3, with no known outcome.
- Three owners acquired a total of six new cats

#### Overall cat data

Of the 63 female cats spayed, 15 (23.8%) were pregnant at the time of spay. Owners reported a total of 54 litters born during their period of cat ownership, with a range of 0–13 and a mean of 5 litters per household. Seven owners agreed that there had been potential inbreeding of cats with one additional owner unsure/confused.

#### Extra revisits

Additional revisits were necessary for Houses 6 and 9, performed 5 and 3 weeks after initial visits respectively. This was in order to spay female cats that had been lactating with young, unweaned litters at the initial visits (1 female in House 6, 2 females in House 9). Other than neutering the 3 female cats, no other activities were conducted at these additional visits.

### Welfare scores (WS)

In House 1, it was not possible to assess a change in WS, as the cats were all relinquished before the 2 month revisit was conducted. Eight of the remaining nine households demonstrated a reduced mean WS at the 2 month revisit (Table 2).

**Table 2:** Overall means of all household Welfare Scores (WS) at each time point, and measures of data distribution and normality

	Overall mean of all Household WS (standard deviation)	Shapiro-Wilk test statistic (*p*-value)
Visit 1	6.6 (1.6)	0.9 (0.4)
Visit 2 (2 months)	4.8 (1.6)	0.9 (0.6)
Visit 3 (3 months)	4.5 (0.9)	0.9 (0.8)

Overall, the difference in mean household WS changed significantly over the study period (F(2,12) = 14.5, *p* = 0.01). Overall the mean household WS values were significantly improved at the 2 month revisits when compared to the initial visits (*p* = 0.011). Although in the remaining households, overall WS values were improved at 12 months when compared with the 2-month visit, this improvement was not statistically significant (*p* = 0.091). All of the households demonstrated a significantly reduced mean WS value at 12 months when compared to the initial visits (*p* = 0.01).

It was not possible to recheck 19/176 (10.8%) of the cats over the 2 revisits, as the owners were unable to secure them inside.

### Owner information

As can be seen in Table 3, many of the participants were experienced cat owners. Three of the owners were currently employed, four were retired, two were full time carers and one was a student. In six of the households, there was a history of past or present mental illness. Two of the ten participants were classified as rescue hoarders, six were overwhelmed caregivers, and two owners did not clearly fit into any one category.

**Table 3:** Assessment of demographic characteristics, cat acquisition styles, cat owning experience and basic cat husbandry facilities of participating households

Multi-cat house number	1	2	3	4	5	6	7	8	9	10
Number of years cat ownership	5	9	19	5	30	6	5	15	2	10
Number of people living in house	5	2	1	1	2	1	2	3	2	2
Gender of people in house Male (M) Female (F)	M,F	M,F	M	F	M,F	M	M,F	M,F	M,F	M,F
Age range, in years, of people in house	13–45	22–48	67	29	22–49	71	59–90	22–52	54–64	52–80
Any history of mental health disorders? (Y/N)	N	Y	Y	Y	Y	N	N	Y	Y	N
Acquisition of cats? Active/Passive/Both (A/P/B)	P	A	P	A	A	B	P	P	P	B
Other animals? (Y/N)	Y	N	N	N	Y	N	Y	N	N	Y
Feed cats: Inside/Outside/Both (In/Out/B)	In	B	In	In	In	In	In	In	In	In
Number of litter trays	3	6	0	4	5	3	3	2	1	4
Number of cats per litter tray at Visit 1	3.7	2.8	NA	1.8	3.3	3.3	11	7.5	21	5.3
Total number of rooms	7	6	4	7	6	5	5	6	4	5

Although 6/10 participants reported that their cats had at least some outside access, only one had a cat flap. Six of the participants reported that they had never taken any of their cats to see a VS, with 82% of the cats in the study never having visited a vet. Reasons cited for taking cats to a vs included initial vaccinations, neutering and road traffic collisions.

Of the participants, 8/10 reported that none of their cats had been previously vaccinated, and two reported that some of their cats had been vaccinated on at least one occasion (10% of the cats in the study). Four and seven participants had provided some cats with occasional, shop bought treatment for worms and fleas respectively. The age range of people living in the multi-cat houses was 13–90 years. The mean age of the individuals identified as having overall responsibility for the cats was 56 years of age.

### Environmental assessments

Most of the environmental scores were relatively low, with no houses having human waste evident and only one showing signs of hoarding of other objects (in this case furniture), which prevented safe movement within the home (Table 4). However in six households there was overflowing rubbish, in seven there were overflowing litter trays and in four animal faeces/urine were noted in areas away from litter trays.

**Table 4:** Assessment of environmental characteristics in participating households.

Multi-cat house number	1	2	3	4	5	6	7	8	9	10
Presence of spoiled food - human	DK^a^	DK	Y	DK	N	Y	N	N	N	N
Presence of spoiled food - animal	DK	DK	N	N	N	N	N	N	N	Y
Is the owner unable to prepare food?	DK	DK	N	DK	N	DK	N	N	N	N
Presence of insects/rodents	N	N	N	N	N	N	Y	N	N	N
Is the owner unable to access the toilet?	DK	DK	N	DK	N	DK	N	N	DK	DK
Is the owner unable to sleep in a bed?	DK	DK	N	DK	N	Y	N	N	DK	Y
Presence of faeces/urine - human	N	N	N	N	N	N	N	N	N	N
Presence of faeces/urine - animal	N	N	Y	Y	N	Y	N	N	N	Y
Presence of mould or chronic dampness	DK	DK	Y	DK	N	DK	N	N	DK	DK
Rubbish overflow	Y	N	N	Y	N	Y	Y	N	Y	Y
Litter trays overflowing	Y	DK	Y	Y	N	Y	Y	N	Y	Y
Hoarding of other objects	N	N	N	N	N	Y	N	N	N	N
Is the owner unable to move freely/safely within the home?	N	N	N	N	N	Y	N	N	N	N
Score /13	2	0	4	3	0	7	3	0	2	5

*Y* Yes, *N* No ^a^*DK* Don’t know (in these households it was not possible to assess all areas of the house)

## Discussion

In this pilot study, ten households were identified that had significant numbers of cats with compromised welfare and uncontrolled breeding. They were owned by people who were unable to meet minimum standards of care, in environments that were compromised.

The overall welfare of the cats remaining within the households significantly improved over the 12 month study period when compared to the initial visits. Although the main intervention was neutering of entire female cats by the VS, it is likely that the improved scores were a consequence of a combination of factors. These factors may include the voluntary reduction in the number of cats by most owners, the sustained relationship with the VS providing ongoing opportunities for education, discussion and continued motivation for the owner to co-operate.

Overall there was a large reduction (40%) in the number of cats over the study period, suggesting this approach may offer promise in tackling multi-cat households by reducing numbers to more manageable levels. It is important to note that this reduction in the number of cats was not imposed on owners. Instead, the owners voluntarily signed animals over, after discussing with the VS how many cats they felt that they could cope with, meaning the reduction in numbers was always owner-led. Perhaps surprisingly, 80% of the owners felt they were not currently coping with the number of cats and choose to sign cats over to the RSPCA. There is no previous literature that appears to assess feline welfare scores in hoarding situations, linked to veterinary interventions in situ. Previously, approaches have often focussed on mass confiscations of affected animals, with or without legal action. This strategy may relieve animal suffering in the short term, but recidivism has been reported to be almost universal [12, 14, 29] . It is possible that a more owner-led approach may lead to a more sustained improvement; however this must be explored in future follow-ups to households to see if the effects of this intervention are sustained.

A relatively high proportion of the cats signed over (25/45, 47%) were later euthanased for welfare reasons. This relatively high proportion of euthanasias is perhaps due to owners signing over the cats that were in the poorest condition, following discussion with the VS. The VS explained to the owner which cats required ongoing and potentially costly medical treatment. This is likely to have influenced the owner’s decision regarding which cats they chose to sign over.

At the initial visits, 80% of the cats were found to be entire. None of the owners had taken their cats for neutering, yet all of the owners in this study consented to having all of their entire female cats (> 8 weeks old) spayed. This suggests that none of the owners were opposed to the concept of neutering itself, or that if they were, this attitude changed following discussion with the VS. It has been suggested that lack of owner understanding around feline reproduction is instrumental in delayed neutering and production of accidental litters [30, 31]. For this multi-cat study, discussions between the owners and the VS at initial visits are likely to have dispelled some of the myths and fears surrounding neutering, perhaps explaining the 100% uptake of spaying on offer. The high rate of pregnant cats being neutered highlights the importance of prompt neutering of all female cats.

Over their period of cat ownership, the ten owners reported a total of 54 litters passing through their households. Assuming a mean litter size of four kittens, this equates to approximately 216 kittens. This is particularly relevant when considering the high volume of cats relinquished to shelters each year in the UK [32]. These households not only need to be considered for the number of cats that they currently own, but also for their potential as contributors to the local owned and stray feline population that is most likely already saturated.

Previous research, examining the outcomes of 56 animal hoarding cases, suggested that more rapid identification of offenders as hoarders and more creative interventions involving long-term monitoring and inclusion of ancillary services could speed resolution of cases [14, 26]. The veterinary interventions in these documented hoarding cases were limited to examining animals and preparing written reports to be used as part of criminal prosecutions. This was an end stage, reactive involvement and differed significantly when compared to the earlier veterinary involvement employed in this study. The VS in this study aimed to work with owners in a cooperative and preventative manner, to hopefully prevent conditions deteriorating to the point that prosecutions may be deemed necessary. None of the households involved in this study had progressed to prosecutions at the time of writing.

The mean age of the main cat carer (56 years), was broadly similar to other reports [6, 16]. Seven of the ten households had both male and female occupants contrasting with existing studies suggesting that animal hoarders are predominantly female [11, 13, 16, 33]. However, we cannot be sure whether the person primarily responsible for the hoarding in such cases was male or female, whether both partners were active participants in hoarding or one or other simply acquiesced. Six of the ten households included individuals with a reported history of past or present mental health disorders. In England an estimated 17% of people report experiencing a mental health problem in any given week [34]. It is difficult to assess whether mental health disorders were overrepresented across the sample households due to the small sample size; animal hoarding has been linked with a number of mental disorders, but there is a lack of consensus on whether there is a consistent underlying pathology [11, 20, 28].

Most of the participating study owners had never taken any of their cats to see a vet, and the majority of the cats had never received any vaccinations or worming treatment. Seventy per cent of the owners had bought flea treatments for their cats at least on some occasions, usually over the counter products. These figures highlight just how difficult it can be as a veterinary profession, to reach this demographic, which potentially represents the most vulnerable cross section of cats.

The majority of our study demographic fitted the criteria for overwhelmed caregivers, with fewer rescue hoarders and no exploiter hoarders noted. The nature of the referral process for this UK study naturally filtered any exploiter hoarders from the cohort, as they were far more likely to have been selected by the referring RSPCA inspectors to feed into an alternative prosecution route. This may account for the lack of representation of the exploiter group.

Whilst certainly of concern, environmental conditions in the households described here were generally less extreme than some of those described in previous reports [23]. For example, one study described human and animal faeces piled so deep “(the) floors buckled” and identified that over half of the households described had impaired access to a toilet or washing facilities, and over three quarters lacked an appropriate place to prepare food or wash [35]. However, the extent to which the human living environment is affected appears to vary depending on the species kept and the style of husbandry, as well as the point in time at which external intervention occurs [24]. It is likely that the households in this study may represent an earlier point in the progression of failing care, than some of those previously described. This may account for the fact that of the ten households described, where it was possible to perform assessment, most had accessible basic facilities such as a toilet, washing and food preparation area. However, most still had overflowing rubbish and litter trays present, and inadequate litter tray provision for the cats.

Improving the welfare in multi-animal environments has been previously identified as an intractable challenge. A number of components previously identified as key in instigating behaviour change have been utilised in the present study. For example, provision of individualised feedback on progress was found to encourage compliance with recycling [36], and this concept was incorporated into the Welfare Scores. Emphasising the benefits to their animals has also been shown to increase engagement and compliance with management interventions [37, 38]. Removing barriers and encouraging owners’ beliefs that they can implement change have also been identified as important aspects in enabling positive behaviour changes [39]. The COM-B (Capability, Opportunity, Motivation) model of behaviour change has been previously utilised to inform an intervention to encourage community neutering of feral cats [40]. This model is applicable to the present study, where the RSPCA’s veterinary and Inspectorate teams aimed to fulfil these principles and empower the study participants to be engaged in improving their cats’ welfare.

### Limitations

It was often not possible to view all of the rooms in the house and so completion of the environmental assessment may not be fully reflective of the cats’ entire environment. Some owners requested that the VS stayed in a particular area such as the front room, in such cases it was not possible to view all of the areas that the cats had access to. It may be that in cases where the VS was restricted to a certain area, the conditions in the other areas were poorer in terms of hygiene or clutter. There is also therefore the possibility that some cats were not viewed. The environmental assessment was only conducted at the initial visit and not at subsequent revisits due to logistical constraints. The fact that it was not repeated, to determine if there was an improvement in the environmental conditions over the 1 year study period, poses a significant limitation.

There was an issue of compliance with difficulty contacting one owner (House 5) to arrange the revisit. This resulted in a revisit date that was delayed by 2 months. It is possible that the additional visits that were performed to spay previously lactating female cats in Households 6 and 9, may have affected subsequent Welfare Scores. This is because it provided an additional point of contact with the VS compared to the other households. However, as no other activities were performed at these extra visits, it is unlikely to have had had a substantial influence on subsequent Welfare Scores.

Additionally, owner responses to questions regarding issues which they may have perceived as sensitive, may have been subject to response bias. It is possible that once a relationship had been established with the VS, the owner subsequently gave answers that they felt would please the VS. For example, when asked whether they had provided preventative parasitic treatments between VS visits, the answers given were not verified further. Perhaps in future studies it would be beneficial to ask owners to keep proof of purchase of such treatments to allow more accurate verification of interim preventative care.

Case definition and method of recruitment provided significant limitations to this study. It was left to RSPCA inspectors to decide which households they referred to the scheme depending on their previous experiences of the household. For example, one owner was referred with ten cats but over the previous 2 years was known to have had up to 30, prompting referral. Where welfare was particularly bad, RSPCA inspectors may have chosen alternative routes such as confiscation and prosecution, and therefore it is likely that this study population omitted the most severely affected households.

This study has focused on feline welfare scores as a marker of success for the veterinary interventions, however the WS is an incomplete assessment, as it does not include aspects such as behaviour and stress, and has not been externally validated. The owners’ mental health conditions were self-reported, and it is possible that due to the sensitive nature of this information, the prevalence may have been under-reported.

## Conclusions

In this study, veterinary home based interventions, primarily spaying female cats, in multi-cat households were successful in improving welfare scores over a 1 year period. In addition, there may be a role in reducing numbers of cats to a more manageable level. This study suggests that partnering with owners of ‘problem’ multi-cat households, as identified by the RSPCA inspectorate, and establishing a relationship of trust rather than punishment, offers promise as a method of preventative intervention for this vulnerable cohort. However, further work is required to assess whether this improvement is sustained over a longer time period, and also whether there are specific types of owner for whom this approach is more likely to be successful. The authors suggest that future collaborative work with other agencies such as mental health charities, social services and environmental health organisations would be of benefit to develop a more integrated and sustained resolution programme.

## Methods

### Case definition

**Fig. 1:**
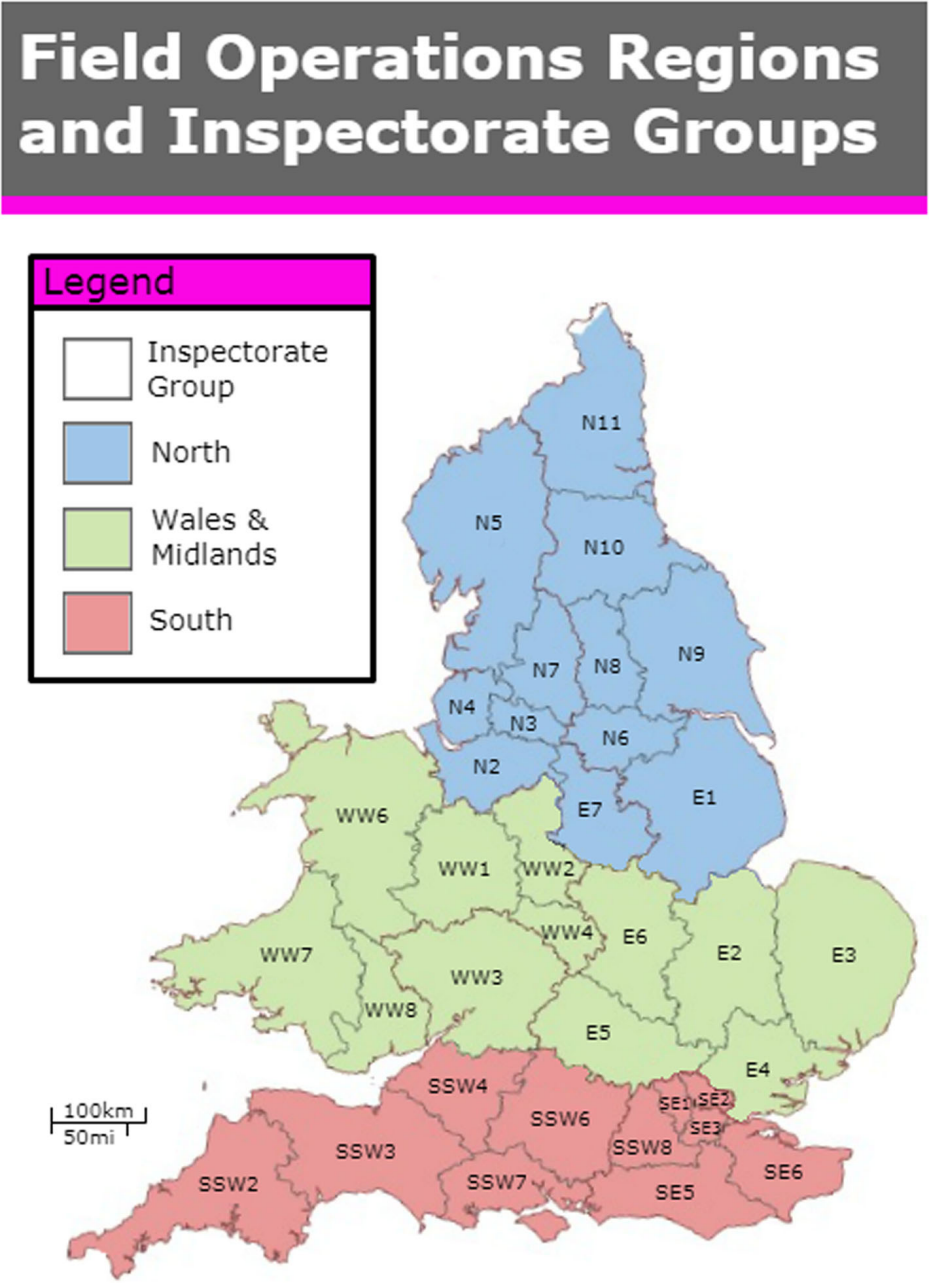
Map showing Inspectorate group regions. N2, N3, N4 and N7 took part in the study. Used by kind permission from the RSPCA

Cases were identified by RSPCA Inspectors from four local inspectorate groups shown in Fig. 1. RSPCA Inspectors were asked to submit details of multi-cat households that they had visited on at least one occasion, following a public complaint passed on through the RSPCA National Call Centre. In order to qualify, households were to have five or more cats of a breeding age (> 4 months), with at least one entire female.

### Recruitment

The designated study veterinary surgeon (VS) (KH) then visited the first ten households, in the order that they were referred by RSPCA inspectors. The RSPCA inspectors advised the owners to expect a call from the RSPCA VS and gave owners a handout explaining the process and interventions offered. The VS then contacted the owner to arrange a date to attend an initial visit to the household. It was explained to the owners that a further two visits would be made after two and 12 months.

### Development of assessment tools

A number of assessment tools were developed to assess the cat population, the individual cats’ health, the history of cat ownership, and the household environment.

#### Cat population data

A data collection form was used to gather information on the overall cat population. This included the number of cats, age, sex, neuter status, microchip status, duration of possession, number of previous litters, whether pregnant or lactating female cats were present and any previous history of veterinary care or preventive medicines such as vaccines or parasitic treatments.

#### Individual cat health – welfare score system

**Fig. 2:**
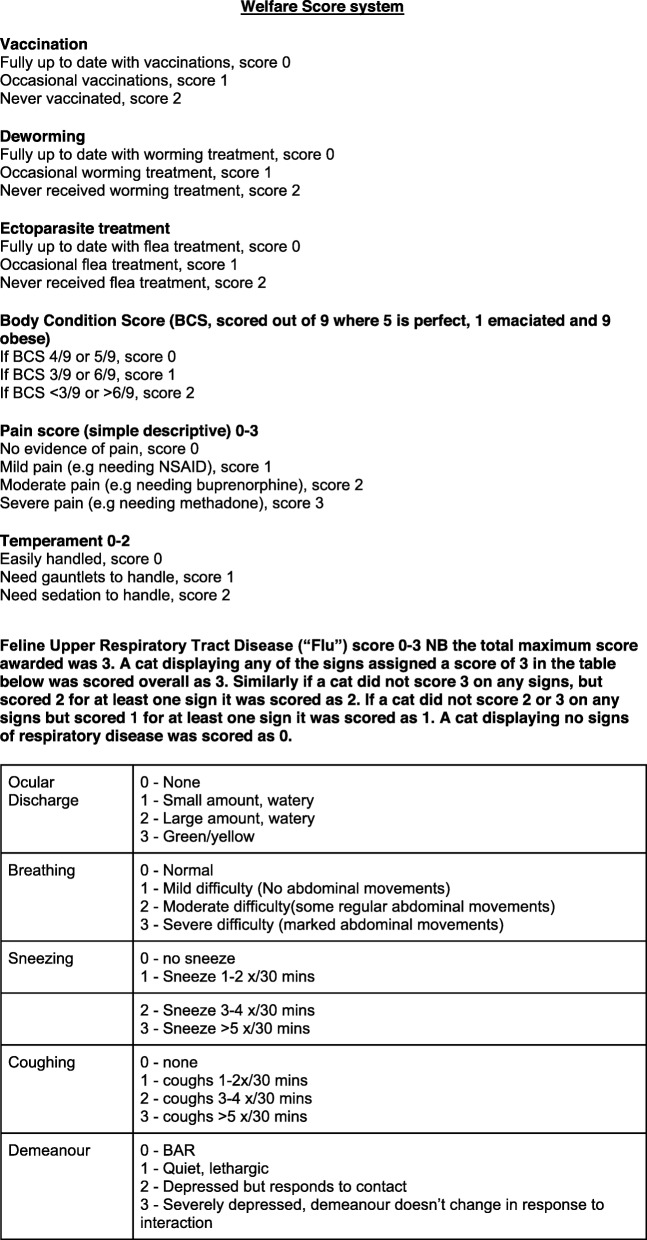
Assessment of basic parameters to form the Welfare Score (WS) for each cat

Each cat was subjected to a scoring system for a combination of clinical parameters. These included Body Condition Score on a 9-point scale [41], simple descriptive pain score, and temperament (score 0 if easily handled through to score 2 if required sedation to handle). Upper respiratory tract disease (“flu score”), was based on an existing system [42]), but simplified for practicality of use in multicat households, and gave an overall score from 0 (no signs of upper respiratory tract disease) to 3 (at least one sign of severe upper respiratory tract disease). The combined scores then gave a numerical welfare score from 0 to 16, with a lower number indicating better overall welfare (Fig. 2). This score was named the Welfare Score (WS). The WS for each individual cat were combined to create a mean household WS for each visit. A simplified version of the WS was created, using traffic light colours instead of numerical values, to provide a summary for each household which was accessible for the owners. This can be summarised as follows -.

Green = good general health.

Amber = may have a health problem but welfare is not immediately impaired.

Red = requires urgent veterinary attention.

#### Ownership information

An owner questionnaire was developed, with 25 questions including number of years of cat ownership and how cats were acquired. Passive acquisition was defined as obtaining cats through breeding within the household. Active acquisition was defined as owners actively seeking to obtain new cats, for example through social media, newspaper advertisements, and rescue shelters. Owners were asked if a second generation of female cats had become pregnant within the household. In such cases, it was noted that there had been the potential for inbreeding. Questions about how long the participants had owned their cats, how they managed their cats’ husbandry (number of litter trays, feeding areas, indoor/ outdoor access) and whether they felt the cats had any visual signs of illness were included.

Owners were also asked if any members of the household had a history of mental health disorders. Owners were categorised by the VS as either overwhelmed caregivers, rescue hoarders or exploiter hoarders based on characteristics described in previous literature [19].

### Environmental assessment

An environmental assessment was based on the existing HOMES (Health, Obstacles, Mental Health, Endangerment, Structure and Safety) Scale [43, 44]. This is a tool that requires rapid initial assessments of multiple issues, to be made in the home environment, based on a visual scan and conversation with the owner.

For the modified study environmental tool, a total of thirteen points could potentially be assigned to observations such as ‘presence of spoiled food - animal’ and ‘litter trays overflowing’ when observed, with a higher overall score indicating a poorer environmental state.

#### Data collection – initial visit

Owners were asked to keep all of their cats indoors from the evening prior to the visit and not to feed the cats on the morning of the visit, in case anaesthesia was required.

At the initial visit, the VS completed the four assessments as described above, and additionally:

1. Microchipping of all cats present
2. Basic health checks on all cats present
3. Transportation of all entire female cats (> 8 weeks of age) to the RSPCA Greater Manchester Animal Hospital. All cats with an American Society of Anaesthesiologists (ASA) Physical Status Scale score of < 2 underwent ovariohysterectomy and full clinical examination under general anaesthesia [45]. Cats with an ASA score of > 2 were discussed individually with the owners, all of whom consented to having these cats humanely euthanased by veterinary professionals.

Each owner was given a handout explaining the cat WS system in layman’s terms, which was verbally explained to any members of the household present.

The following day, all spayed female cats were returned to the owner. The VS discussed a summary of the clinical findings and welfare scores for the owner’s cats alongside education on vaccinations, flea and worming treatment. Written advice including targeted goals (for example when to next apply a flea treatment) was provided as an additional resource for owners. If any individual cats required medical treatment, this was discussed and medication was dispensed as necessary, with advice on seeking further veterinary treatment.

### Data collection – revisits

At the planned revisits 2 and 12 months later, the VS completed the following -

1. Cat population data assessment
2. Basic health checks on all cats present
3. WS for each individual cat present
4. Microchipping of any new cats or cats missed at the initial visit

At each of the visits, the VS asked the owner whether they felt that they were coping with the current number of cats. If the owner felt that they were not coping, they were given the option to reduce numbers by signing some of the cats over to the RSPCA.

### Statistical analysis

Results were stored securely in Google sheets. Descriptive and inferential statistics were calculated in IBM SPSS Statistics for Windows (Version 24.0. Armonk, NY: IBM Corp.). The distribution of mean household WS at each time point was checked for normality using the Shapiro-Wilk test and compared over the three visits using Related-Measures ANOVA. Mean household WS was then compared between pairs of time points using the two-tailed Paired Samples student’s t-test. A *p*-value of < 0.05 was considered as significant throughout.

## Data Availability

The datasets used and/or analysed during the current study are available from the corresponding author on reasonable request.

## Abbreviations

A: Active acquisition of cats
ASA: American Society of Anaesthesiologists
B: Both active and passive acquisition of cats
BCS: Body Condition Score
DK: Don’t know
F: Female
GMAH: Greater Manchester Animal Hospital
HOMES: Health, Obstacles, Mental Health, Endangerment, Structure and Safety Scale
In: Inside
IQR: Interquartile range
M: Male
N: No
Out: Outside
P: Passive acquisition of cats
VS: Veterinary Surgeon
WS: Welfare Score
Y: Yes
